# Profiling Blood-Based Neural Biomarkers and Cytokines in Experimental Autoimmune Encephalomyelitis Model of Multiple Sclerosis Using Single-Molecule Array Technology

**DOI:** 10.3390/ijms26073258

**Published:** 2025-04-01

**Authors:** Insha Zahoor, Sajad Mir, Shailendra Giri

**Affiliations:** Department of Neurology, Henry Ford Health, Detroit, MI 48202, USA; smir6442@gmail.com

**Keywords:** biomarker, cytokines, glial fibrillary acidic protein (GFAP), neurofilament light chain (NFL), single molecule array (SIMOA), experimental autoimmune encephalomyelitis (EAE), multiple sclerosis (MS)

## Abstract

Experimental autoimmune encephalomyelitis (EAE) is a preclinical animal model widely used to study multiple sclerosis (MS). Blood-based analytes, including cytokines and neural biomarkers are the predictors of neurodegeneration, disease activity, and disability in patients with MS. However, understudied confounding factors cause variation in reports on EAE across animal strains/studies, limiting the utility of these biomarkers for predicting disease activity. In this study, we investigated blood-based analyte profiles, including neural markers (NFL and GFAP) and cytokines (IL-6, IL-17, IL-12p70, IL-10, and TNF-α), in two clinically distinct EAE models: relapsing-remitting (RR)-EAE and chronic-EAE. Ultrasensitive single-molecule array technology (SIMOA, Quanterix) was used to profile the analytes in the blood plasma of mice at the acute, chronic, and progressive phases of disease. In both models, NFL was substantially increased during post-disease onset across all phases, with a pronounced increase observed in chronic-EAE. The leakage of GFAP into peripheral blood was also greater after disease onset in both EAE models, especially in the acute phase of chronic-EAE. Among all cytokines, only IL-10 had consistently lower levels in both EAE models throughout the course of disease. This study suggests NFL, GFAP, and IL-10 as potential translational predictors of disease activity in EAE, making them potential candidates as surrogate markers for the preclinical testing of therapeutic interventions in animal models of MS.

## 1. Introduction

Multiple sclerosis (MS) is an inflammatory disease of the central nervous system (CNS) characterized by immune-mediated damage to the insulating myelin sheath, resulting in progressive demyelination and neurodegeneration. Experimental autoimmune encephalomyelitis (EAE) is a well-established preclinical animal model of MS [1,2,3,4,5]. Despite certain inherent limitations, the development of potential treatment options for MS has improved [6,7]. EAE represents a complex heterogeneous model that relies on the disease induction method used, which adds variability to disease pathogenesis, making it an imperfect but approximate model for studying MS. It is worth mentioning that its use depends entirely on the scientific question being addressed and the feasibility of translation. There are two widely used EAE models that approximate the relapsing-remitting (RR) and chronic disease phenotypes of MS: the SJL-based biphasic RR-EAE model, and the monophasic B6 chronic model [8,9,10,11]. The disease is induced in EAE through an immune-mediated response to CNS components by active immunization with self-antigens to myelin, making it an inflammatory model [12,13]. The CNS antigens targeted are myelin basic protein (MBP), myelin oligodendrocyte glycoprotein (MOG), or proteolipid protein (PLP). EAE, as an animal model, is considered the backbone for preclinical predictions and testing potential drugs for MS by monitoring effects on histopathology, motor deficits, and inflammatory responses in EAE [12]. Therefore, it continues to be the most appropriate and relevant animal model to study MS since its inception 90 years ago [7].

EAE is characterized by impaired inflammation resolution accompanied by the dysregulation of cytokine profiles, immune cell infiltration into the CNS, disruption of the blood–brain barrier (BBB), neuroinflammation, and neuroaxonal pathologies, including demyelinating lesions and gliosis [14]. Although the precise mechanism of EAE pathogenesis is still unclear, the pathogenic cascade mainly involves the production of cytokines by immune cells throughout the disease course, ultimately compromising BBB integrity and tissue damage [12]. Measuring blood-based markers is developing importance in assessing the disease activity of MS and its animal model EAE [15]. Effective biomarkers for MS diagnosis, disease progression, and treatment responses, particularly those measurable in the blood, are sorely lacking. While significant effort has been made to identify biomarkers from cerebrospinal fluid (CSF) to diagnose MS, this endeavor has been challenging with limited success [16]. The analysis of easily drawn biofluids, including blood for MS biomarkers, has been minimally investigated but holds significant promise. Neurofilament light (NF-L), glial fibrillary acidic protein (GFAP), and cytokines (IL-17, IL-6, IL-10, TNF-α, IFN-γ, and IL-12) are emerging biomarkers for determining inflammatory disease activity, disease progression, treatment response, and prognosis in MS patients [17,18,19,20,21]. The leakage of NFL and GFAP into plasma/serum is particularly useful for monitoring neuroaxonal damage in MS, and yet there are limited studies on this topic in the preclinical animal models of EAE [17,22,23,24].

Given the urgent need for blood-derived biomarkers for MS, the inception of highly sensitive immunoassays serves as the foundation. Conventional methods of measuring blood biomarkers have certain limitations due to low sensitivity and inconsistency across reports showing the profiles of neural markers and cytokines in preclinical models [25]. Comparisons across profiling studies of EAE often lack reproducibility due to variability across mice and underlying confounding factors such as the extent of inflammation or demyelination and small sample sizes [22]. The biological matrix used for profiling blood-based analytes also contributes to variability.

With the increase in neuroprotective or reparative strategies, potential therapeutics for EAE must be evaluated through quantitative neuropathology. Herein, we applied SIMOA (SIngle MOlecule Array), as this is an emerging ultrasensitive technology based on a bead–conjugate immunocomplex with the potential to detect analytes present at ultralow levels (femtomolar range) and below detection limits than other conventional assays [26,27,28,29]. The average number of enzymes per bead (AEB) is considered the unit of measurement. Several different biomarkers can be detected in a single experiment, in a variety of sample types, with singleplex and multiplex assays. According to Quanterix, this digital platform can precisely measure analyte levels in human blood samples with 1000 times greater sensitivity than conventional methods like (enzyme-linked immunosorbent assay) (ELISA) with the ability to quantify proteins that are far lower than the Level of Quantification (LoQ) (https://www.quanterix.com/simoa-technology/; accessed on 25 December 2023). This means that subtle changes can be captured earlier and less invasively compared to other approaches [30]. In this study, we aimed to further investigate the outcome of EAE manifestations in the form of disease severity parameters and the detectability of plasma NFL, GFAP, and cytokines over the course of the disease in two models of EAE. Overall, we propose the application of SIMOA as a valuable approach for the indirect assessment of disease progression and pathology in EAE which is crucial for understanding of neurodegeneration and neuroinflammation in MS.

## 2. Results

### 2.1. EAE Increases NFL and GFAP Leakage in the Peripheral Circulation

**Figure 1:**
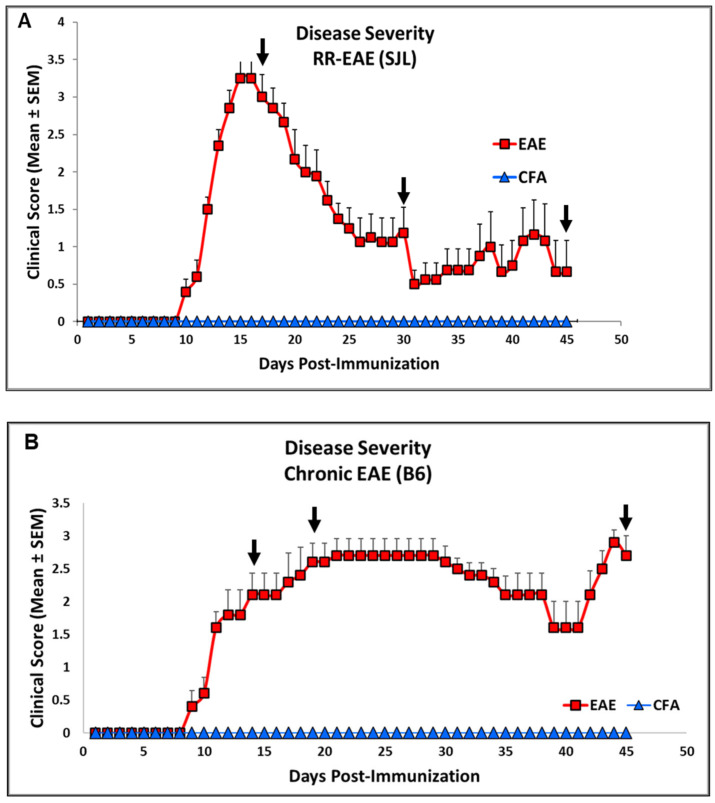
Representative clinical scores as indicators of disease severity changes in RR (SJL) and Chronic (B6) models of EAE. Clinical score in SJL (**A**) and B6 mice (**B**) consisting of experimental groups CFA and EAE during disease course. Arrows indicate the time points used for profiling the analytes. Scores are shown as mean ± SEM.

**Figure 2:**
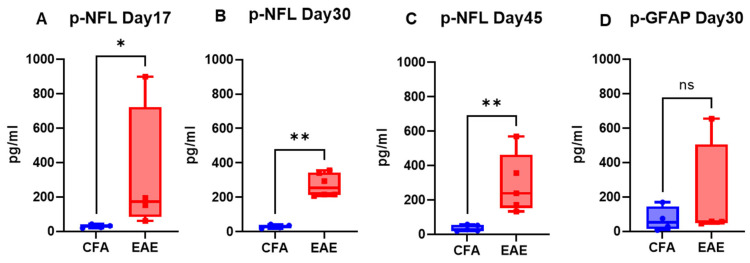
Profile of neural markers (NFL and GFAP) in plasma of RR-EAE. Plasma NFL (**A**–**C**) and GFAP (**D**) levels at respective time points in CFA vs. EAE. Values are shown in pg/mL. * *p* < 0.05, ** *p* < 0.01, ns non-significant (as determined by Mann–Whitney *t*-test).

**Figure 3:**
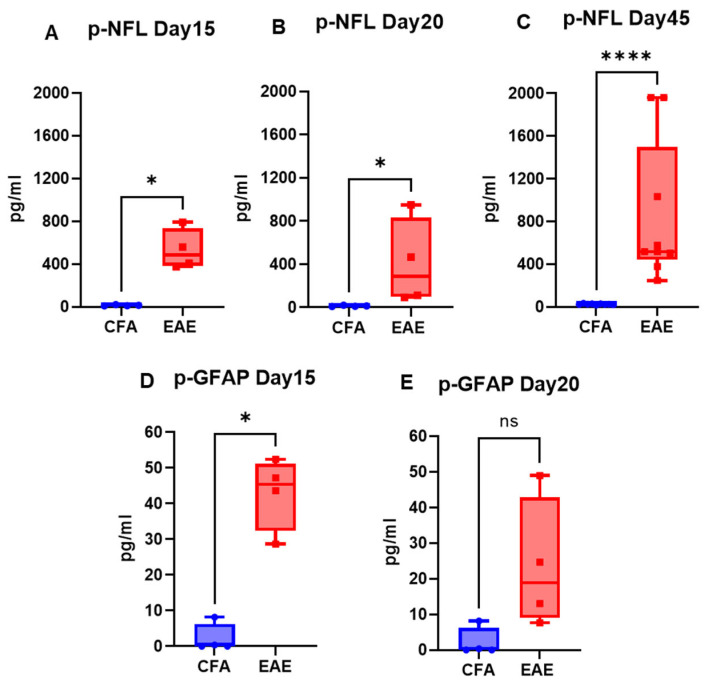
Profile of neural markers (NFL and GFAP) in plasma of Chronic-EAE. Plasma NFL (**A**–**C**) and GFAP (**D**,**E**) levels at respective time points in CFA vs. EAE. Values are shown in pg/mL. * *p* < 0.05, **** *p* < 0.0001, ns non-significant (as determined by Mann–Whitney *t*-test).

**Figure 4:**
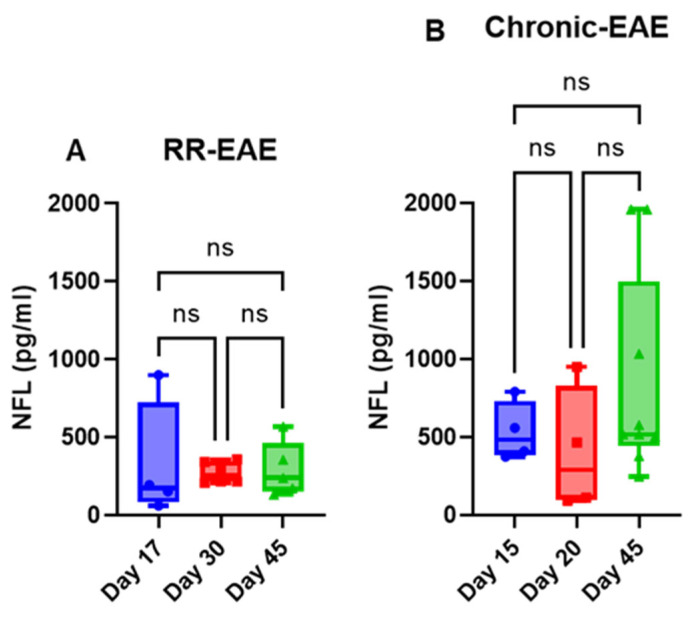
Comparative plasma NFL concentrations along disease duration in RR-EAE (**A**) and Chronic-EAE (**B**). Values are shown in pg/mL as determined by Kruskal–Wallis one-way ANOVA test, CFA vs. EAE, ns non-significant.

The mice injected with respective antigens (PLP_139–151_ and MOG_35–55_) for EAE induction in both RR (SJL) and chronic (B6) models showed classical disease courses with established symptoms which was evident from clinical scores (Figure 1). As expected, CFA control mice did not show any clinical symptoms of disease. We have previously shown that histological analyses of spinal cords show an extensive demyelination and antigen specific response in EAE, whereas control mice do not exhibit signs of demyelination and antigen specific response [31]. In the present study, neuroaxonal damage in the CNS was assessed by measuring the leakage of NFL and GFAP into peripheral circulation over the disease course of EAE. As shown in Figure 2 and Figure 3, compared to those in the control CFA group, plasma levels of NFL and GFAP were significantly higher at all time points in both EAE models. The mean NFL level for CFA mice in the RR-EAE model was 31.68 ± 1.93 pg/mL compared to EAE mice, with an average value of 297.45 ± 15.77 pg/mL across all time points when combined (Figure 2). During the peak phase of the disease on day 17, compared to CFA mice, the mean NFL level was 326.42 pg/mL (*p* < 0.05; median 173 pg/mL, clinical score ~3–4) (Figure 2A) in the induced RR-EAE model, and was greater than other time points (day 30, 272.15 pg/mL, *p* < 0.01, median 255.6 pg/mL; day 45, 298.8 pg/mL, *p* < 0.01, median 238.4 pg/mL) (Figure 2B,C), reflecting a substantial increase compared to control mice, and clearly highlighting the pathological manifestations of EAE (Figure 2). Overall, NFL levels ranged between 60.43 and 899.25 pg/mL in the RR-EAE model across all time points compared to CFA, which was between 18.35 and 57.75 pg/mL, suggesting the elevated leakage of NFL in EAE (Table 1). Furthermore, the mean NFL level for the entire disease course reached 598.11 ± 133.83 pg/mL in the chronic-EAE model compared to 17.77 ± 4.08 pg/mL in CFA mice, with time-specific mean values of 533.5 pg/mL in EAE mice on day 15 (clinical score ~3–4) (*p* < 0.05; median 484.3 pg/mL), 405.48 pg/mL on day 20 (*p* < 0.05; median 289.8 pg/mL), and day 45 (mean value 855.37 pg/mL; *p* < 0.0001; median 517.4 pg/mL) (Figure 3). Overall, NFL values in chronic-EAE models ranged between 92.47 and 1960.59 pg/mL, whereas NFL values in CFA mice ranged between 9.53 and 34.77 pg/mL (Table 1). By and large, there was no significant difference in NFL levels across disease stages in both RR-EAE and chronic-EAE (Figure 4).

While the mean plasma level of GFAP in the RR-EAE group was 203.56 ± 173.60 pg/mL (median 57.04 pg/mL), it was 69.88 ± 41.37 pg/mL (median 51.82 pg/mL) in the CFA mice of this model, suggesting that leakage in the EAE group was still significant (Figure 2D). The range of GFAP levels was 45.65 to 654.52 pg/mL in RR-EAE compared to 7.22 to 168.70 pg/mL in CFA mice (Table 1). For the chronic-EAE model, when compared across all time points, the average plasma level of GFAP was 33.27 ± 7.92 pg/mL compared to 2.14 ± 0 pg/mL for CFA. Leakage reached a maximum on day 15 (*p* < 0.05; median 45.43 pg/mL), with an average value near 42.98 pg/mL compared to day 20 with 23.56 pg/mL (median 18.85 pg/mL) in the EAE group (Figure 3D,E). The detection range for GFAP in the chronic-EAE model was 7.62 to 52.40 pg/mL in the EAE group compared to 0.00 to 8.17 pg/mL in the CFA group (Table 1). Taken together, NFL and GFAP showed substantial leakage from the CNS into the periphery in both models of EAE post-disease immunization.

### 2.2. EAE Decreases the Level of the Anti-Inflammatory Cytokine IL-10 in the Blood

**Figure 5:**
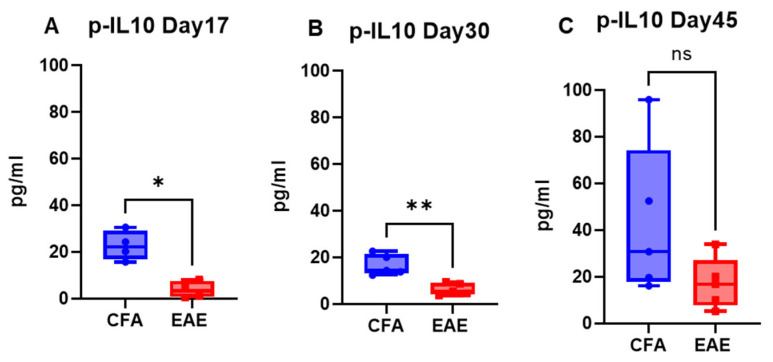
IL-10 profile in plasma of RR-EAE. Plasma level of IL-10 at respective time points (**A**–**C**) in CFA vs. EAE. Values are shown in pg/mL. * *p* < 0.05, ** *p* < 0.01, ns non-significant (as determined by Mann–Whitney *t*-test).

**Figure 6:**
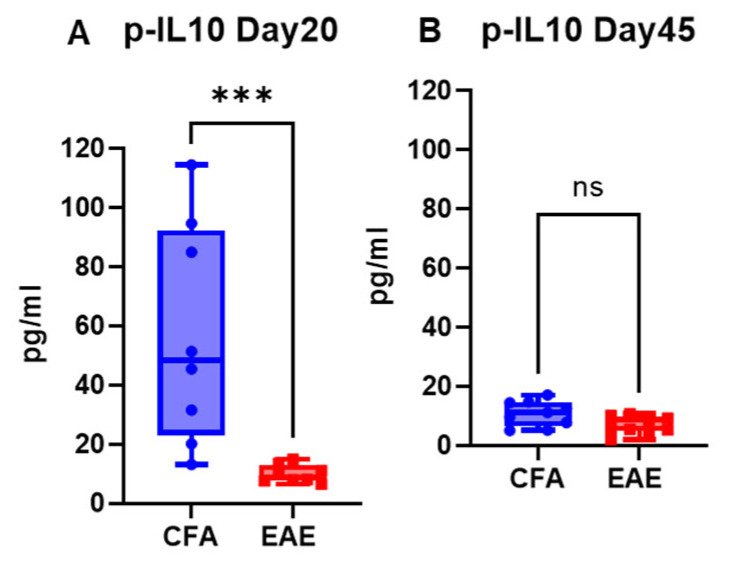
IL-10 profile in plasma of Chronic-EAE. Plasma level of IL-10 at respective time points (**A**,**B**) in CFA vs. EAE. Values are shown in pg/mL. *** *p* < 0.001, ns non-significant (as determined by Mann–Whitney *t*-test) vs. EAE.

IL-10 plays a crucial role in the pathophysiology of MS and EAE animal models because it is a key cytokine involved in regulating the immune response [32]. In the present study, the plasma level of IL-10 was significantly lower in the EAE group than in the CFA group, with the mean level of IL-10 in the RR-EAE group approaching 3.76 pg/mL on day 17 (*p* < 0.05; median 3.24 pg/mL) (Figure 5A), 6.35 pg/mL on day 30 (*p* < 0.01; median 5.49) (Figure 5B), and 17.18 pg/mL on day 45 (median 16.77 pg/mL) (Figure 5C). The level of IL-10 was lowest on day 17 (clinical score ~3–4), in the peak phase of disease, compared to other time points (Figure 5). The mean concentration of IL-10 was 9.09 ± 4.11 pg/mL for RR-EAE, compared to 27.43 ± 7.95 pg/mL for CFA, with a detection range of 0.46 to 33.87 pg/mL for EAE and12.38 to 95.88 pg/mL for CFA (Table 1). Similarly, the same downward trend was found in chronic-EAE, with mean IL-10 concentrations of 7.82 ± 1.71 pg/mL in the EAE group and 34.06 ± 18.68 pg/mL in the CFA group, and with detections ranging from 1.92 to 14.80 pg/mL in the EAE group and from 5.07 to 114.40 pg/mL in the CFA group (Figure 6; Table 1). The maximum decrease in the IL-10 concentration in EAE mice was observed on day 45 (mean level 9.92 pg/mL; *p* < 0.001; median 8.91 pg/mL) compared to day 20 (5.73 pg/mL; median 6.74 pg/mL). Overall, IL-10 concentrations decreased in both models of EAE.

## 3. Discussion

The current goal of MS biomarker research is to use sensitive platforms that generate a specific biomarker profile to improve the diagnosis and management of MS. SIMOA technology is a fully automated platform that detects analytes (mainly proteins) when bound to antibody-coated beads combined with high-resolution fluorescence imaging [30,33]. Currently, the two most promising biomarker candidates for assessing neural damage in MS patients are NFL and GFAP; however, results are inconsistent across studies, which is highly suggestive of the variability in techniques used to measure them, as well as the heterogeneity across study cohorts [34]. NFL represents the cytoskeletal component of neurons, while GFAP represents the cytoskeletal intermediate filament of astrocytes, and both serve as biomarkers of neuronal death, axonal degeneration, and astrogliosis [35]. Blood NFL is a neuro-axonal injury marker associated with MS relapse, the worsening of EDSS scores, lesions on MRI scans, and atrophy of the brain and spinal cord in patients with MS [36]. NFL has been investigated as a potential prognostic and disease activity marker, with a potential relationship between its leakage and the rate of neurodegeneration [37]. Blood-based detection of NFL highlights early demyelination, while GFAP is mainly analyzed for MS progression and severity. The ability to measure NFL at different time points in serum/plasma also makes it suitable for monitoring treatment response. However, there are several limitations in the application of NFL detection. As a cytoskeletal protein that can be released by any kind of brain injury, it is not specific to MS; thus, any other neurologic disease or injury can have confounding effects. It is more logical to detect a panel of biomarkers using an ultrasensitive platform that would enhance the specificity, accuracy, and reproducibility of characterizing MS and post-treatment assessments.

The CNS biomarkers NFL and GFAP are used to monitor pathophysiological and neurodegenerative manifestations in preclinical MS and EAE animal models. The present study covers both RR-EAE (SJL) and chronic-EAE (B6) models, contrasting with previously published reports that use only the B6 model [22,23]. Since NFL leakage is associated with impaired BBB integrity, immune cell extravasation, and CNS inflammation following the first demyelinating event in MS [38], we measured its plasma level in both EAE models. Notably, NFL leakage in the EAE model was more pronounced for the chronic-EAE model than the RR-EAE model, with no conclusive trend for kinetics across time points in either model. This contrasts with the findings of Aharoni et al., who reported the greatest leakage to occur at the peak of disease followed by a subsequent decline as the disease progressed [22]. However, this study was underpowered (n < 3) and variable across time points, limiting the significance of their data. Additionally, findings reported by Gnanapavan et al. showed opposite trends in B6 mice, possibly due to either the application of less sensitive non-SIMOA methods for NFL detection, or batch variation in mice [23]. In the present study, neurodegeneration in the form of neuroaxonal damage was confirmed by the substantial leakage of NFL into peripheral circulation on day 17 (mean value 326.42 pg/mL) after disease induction in the RR-EAE model, overlapping with a maximum clinical score of ~3–4 in these mice. As the disease progressed, NFL levels declined in EAE models at other time points (day 30 and day 45). Furthermore, the average level of NFL was greater in the chronic-EAE (B6) model (598.11 ± 133.83 pg/mL) compared to the RR-EAE (SJL) model (297.45 ± 15.77 pg/mL) for the combined time points, with the highest value in B6 nearing 1960.59 pg/mL. These findings further substantiate NFL as a reliable biomarker for assessing disease activity in EAE. However, the dynamics of NFL in the blood are a limiting factor, making it difficult to link NFL leakage with the degree of neurodegeneration and the extent of CNS damage. Similarly, we detected an increase in plasma GFAP after EAE induction in both models, with a maximum increase detected in the RR-EAE group (203.56 ± 173.60 pg/mL). Taken together, our findings for NFL and GFAP confirm the substantial leakage of CNS components into the periphery as a pathological manifestation of CNS damage in EAE.

Moreover, pathogenic cytokines (IFN-γ, IL-17, TNF-α, IL-6, IL-12p70, and IL-10) can also reflect the degree of inflammation in patients, as MS therapies are largely aimed at lowering the inflammatory response by modulating the levels of inflammatory markers [39]. Cytokine profiles have been established in various studies of adult and pediatric patients with MS [20,40,41,42,43]; however, this process is far more complex since it involves an array of cytokines that have temporal effects on MS. Due to this complexity, detecting multiple variables, including CNS damage biomarkers, provides a more meaningful option for monitoring disease evolution. In the present study, the concentrations of cytokines IL-6, IL-17, IL-12p70, and TNF-α were below the detection limit (conc. lower than the lowest calibrator) across all time points in most of the samples, which is why there was no conclusive data, making it difficult to interpret the trend in EAE (Table 1). However, we found a lower level of IL-10 in both models of EAE, with a mean of 9.09 ± 4.11 pg/mL in RR-EAE and 7.82 ± 1.71 pg/mL in chronic-EAE. Given the central role of IL-10 in the pathophysiology of MS and other neurodegenerative diseases, its lower production in humans is considered a risk factor for MS [32,44]. IL-10 also regulates inflammation-mediated CNS damage in EAE, as evidenced by the exacerbation of EAE in IL-10 KO mice, compared to mice overexpressing IL-10 [45,46]. Overall, our findings indicate that IL-10 is suppressed in EAE, confirming its anti-inflammatory role that suppresses MS.

Notably, GFAP and IL-10 were not detected by SIMOA at the other time points mentioned in the study design, as the values for CFA and EAE mice in both models were either zero for GFAP or below the detection limit for IL-10. These readings could be due to machine error, the assay not working for those samples, or issues with bead–analyte binding. Given the sensitivity of the SIMOA platform, sample processing was uniform across all batches, ruling out its impact on the data. Even so, our data indicated that disease severity parameters and the plasma profiles of CNS biomarkers (NFL and GFAP) and IL-10 can be used together to monitor pathological manifestations over disease duration and to test therapeutic interventions in EAE models. These parameters can also be linked with other analyses to provide a comprehensive profile of drug testing in EAE. This finding was confirmed in our study showing a protective effect of the pro-resolution lipid mediator maresin1 (MaR1) in EAE, where we used SIMOA along with CNS-related histopathological data and other immunological parameters to validate this. Compared to those in the untreated group, the plasma levels of NFL in MaR1-treated EAE-induced mice were lower, confirming the neuroprotective effect of MaR1 [47]. Notably, we have also performed SIMOA assays in human patient samples, which again confirmed the use of NFL and GFAP as biomarkers of disease activity in MS, validating the utility of the SIMOA platform from animal models to human samples [48,49]. The present study is the first to report multiple biomarker profiling in plasma throughout the disease course of EAE using an ultrasensitive platform in two separate EAE models [22]. One limitation of our study is its exclusivity to female mice, with a small sample size. However, we have used more than one EAE model (RR and Chronic) to show that any observed effects are pervasive.

## 4. Materials and Methods

### 4.1. EAE Model Induction

A group of 5–10 female SJL/J and C57BL/6 (ten- to twelve-week-old) mice were purchased from the Jackson Laboratory (Bar Harbor, ME, USA). Animals were housed in a pathogen-free animal facility at Henry Ford Health, Detroit, MI, USA, according to animal protocols approved by the Institutional Animal Care and Use Committee (IACUC). All mice were housed with standard food and water ad libitum at a room temperature of 22 ± 2 °C under a 12:12 h light-dark cycle. For the RR-EAE model, SJL mice were immunized on day 0 via subcutaneous injections in the flank region with the antigen PLP_139–151_ peptide (100 μg/mouse) emulsified in 200 μL Complete Freund’s Adjuvant (CFA) (Sigma Chemicals, St. Louis, MO, USA) supplemented with 4 mg/mL heat-killed *Mycobacterium tuberculosis* H37Ra (400 μg; Becton, Dickinson and Company, Sparks, MD, USA) as described previously [31,47,48,49,50,51]. For the chronic model, EAE was induced in B6 mice on day 0 with 200 μL CFA containing the antigen MOG_35–55_ (200 µg/mouse) prepared similarly, as described previously [31,50,51]. Pertussis toxin (List Biological Labs, Campbell, CA, USA) was administered via intraperitoneal (i.p.) injection (200 ng/200 μL) on days 0 and 2. In both models, one set of mice was injected with CFA without antigen/peptide to serve as a control.

### 4.2. Clinical Assessment of EAE Models

For clinical evaluation of the models based on neurological deficits, the animals were monitored daily, and the clinical score was recorded until the duration of the study, in a blinded fashion by observing disease signs, including paralysis according to the conventional grading system on a scale of 0–4, as described previously [31,47,48,49,50,51]. The onset of disease in EAE was considered on the day animals presented the first signs of disease. The disease peak was considered when the EAE score reached its maximum and did not increase from the previous day (16–19 days postimmunization). The chronic phase of the disease was considered after 20 days postimmunization if the EAE score decreased or was maintained, after the disease peak. Weight loss was monitored after EAE induction. Changes in weight during the disease course were evaluated for each mouse. Readings were taken every week. Animals were provided appropriate supportive care and maintenance while showing disease signs. Mice with severe conditions called for immediate premature euthanasia to meet humane endpoint criteria.

### 4.3. Experimental Groups and Blood Sampling

Mice were euthanized at different time points during the disease course. The time points selected for EAE profiling included day 17 (peak), day 30 (chronic), and day 45 (progressive) for RR-EAE (SJL) and day 15 (before peak), day 20 (peak), and day 45 (chronic) for chronic-EAE (B6). Mice were anesthetized with CO_2_ and transcardially perfused with 1X chilled PBS. Control CFA-treated mice were also included at the indicated time points. Blood samples from mice were collected in ethylenediaminetetraacetic acid (EDTA)-containing tubes for plasma analysis. Plasma was separated from blood by centrifugation at 1500× *g* for 10 min. The clear yellow liquid supernatant was collected from the top and stored in respective tubes at −80 °C until further processing (zero freeze/thaw cycles). The secondary method of euthanasia for mice was either cervical dislocation or needle-induced pneumothorax. All animal experiments in this study were performed per the policies and guidelines of the Institutional Animal Care and Use Committee (IACUC) at Henry Ford Health under the animal welfare assurance number D16-00090.

### 4.4. Single Molecule Array (SIMOA) Assay

To characterize the profiles of neural markers and cytokines in EAE, we measured NFL and GFAP in plasma samples from CFA and EAE mice in both models (RR and chronic) with a commercially available SIMOA™ Neuro 2-Plex B Advantage Kit (product number: 103520) (Quanterix, Billerica, MA, USA) on an SR-X analyzer, according to the manufacturer’s instructions. Additionally, cytokine profiling was performed using a mouse Cytokine 5-Plex Kit for IL-6, TNF-A, IL-17, IL-12, and IL-10 (Cat # 107-178-1-AB; Product Number: 85-0441) (Quanterix, Billerica, MA, USA) on an SP-X analyzer, according to the manufacturer’s instructions. Calibrators supplied with the kits were used as standards. The number of mice used for each assay at a given time point was 4–9. Samples were only loaded after centrifugation at 10,000 rpm for 10 min to prevent lipids in the sample from interfering with the assay. For every 96-well plate-based assay, one batch of reagents was used to minimize the lot-specific variations, as emphasized in the protocol instructions by Quanterix.

### 4.5. Data Analysis

Statistical significance was computed by Mann–Whitney *t*-test and all values are presented as the median or mean ± SEM wherever applicable. The figure legends (and wherever applicable) mention statistical significance and power in terms of “n” and *p* values. Comparisons between different time points across disease courses in both models were performed using Kruskal–Wallis one-way ANOVA test. All statistical analyses were performed using GraphPad Prism Software, version 9.2.0 (San Diego, CA, USA; www.graphpad.com). An asterisk indicates statistical significance * for *p* ≤ 0.05, ** for *p* ≤ 0.01, *** for *p* ≤ 0.001, and **** for *p* ≤ 0.0001 with n > 4 for all variables.

## 5. Conclusions

Taken together, our results validate the utility of the SIMOA as a robust analytical platform with high sensitivity that can precisely detect small changes in samples, making it a highly valuable approach in clinical settings for both human patients with MS and EAE animal models. At the same time, inconsistency in the data, variable sample size, and variation across mouse strains/batches due to unknown confounding variables cannot be ruled out, highlighting the use of caution when interpreting data across studies and making associations between these analytes and disease status. However, further large-scale prospective studies are warranted to validate the detection range and utility of these translational biomarkers in clinical practice.

## Figures and Tables

**Table 1:** Comparative detection range of analytes in plasma of RR and chronic models of EAE using SIMOA.

Analyte	Conc Range (SJL) (pg/mL)		Status in EAE	Conc Range (B6) (pg/mL)		Status in EAE
	CFA	RR-EAE		CFA	Chronic-EAE	
**NFL**	18.35 to 57.75	60.43 to 899.25	Increase	9.53 to 34.77	92.47 to 1960.59	Increase
**GFAP**	7.22 to 168.70	45.65 to 654.52	Increase	0.00 to 8.17	7.62 to 52.40	Increase
**IL-10**	12.38 to 95.88	0.46 to 33.87	Decrease	5.07 to 114.40	1.92 to 14.80	Decrease
**IL-6**	Level below low calibrator	Level below low calibrator	Not conclusive	Level below low calibrator	Level below low calibrator	Not conclusive
**IL-12p70**	Level below low calibrator	Level below low calibrator	Not conclusive	Level below low calibrator	Level below low calibrator	Not conclusive
**TNF-α**	Level below low calibrator	Level below low calibrator	Not conclusive	Level below low calibrator	Level below low calibrator	Not conclusive
**IL-17**	Level below low calibrator	Level below low calibrator	Not conclusive	Level below low calibrator	Level below low calibrator	Not conclusive

## Data Availability

All the data generated or analyzed during this study are included in this published article.

## References

[1] Gold R., Linington C., Lassmann H. (2006). Understanding pathogenesis and therapy of multiple sclerosis via animal models: 70 years of merits and culprits in experimental autoimmune encephalomyelitis research. Brain.

[2] Steinman L., Zamvil S.S. (2005). Virtues and pitfalls of EAE for the development of therapies for multiple sclerosis. Trends Immunol..

[3] Steinman L., Zamvil S.S. (2006). How to successfully apply animal studies in experimental allergic encephalomyelitis to research on multiple sclerosis. Ann. Neurol..

[4] Farooqi N., Gran B., Constantinescu C.S. (2010). Are current disease-modifying therapeutics in multiple sclerosis justified on the basis of studies in experimental autoimmune encephalomyelitis?. J. Neurochem..

[5] Bjelobaba I., Begovic-Kupresanin V., Pekovic S., Lavrnja I. (2018). Animal models of multiple sclerosis: Focus on experimental autoimmune encephalomyelitis. J. Neurosci. Res..

[6] Steinman L. (2009). A molecular trio in relapse and remission in multiple sclerosis. Nat. Rev. Immunol..

[7] Steinman L., Patarca R., Haseltine W. (2023). Experimental encephalomyelitis at age 90, still relevant and elucidating how viruses trigger disease. J. Exp. Med..

[8] Baker D., Amor S. (2014). Experimental autoimmune encephalomyelitis is a good model of multiple sclerosis if used wisely. Mult. Scler. Relat. Disord..

[9] Baker D., Amor S. (2015). Mouse models of multiple sclerosis: Lost in translation?. Curr. Pharm. Des..

[10] Amor S., Baker D. (2012). Checklist for reporting and reviewing studies of experimental animal models of multiple sclerosis and related disorders. Mult. Scler. Relat. Disord..

[11] Miller S.D., McMahon E.J., Schreiner B., Bailey S.L. (2007). Antigen presentation in the CNS by myeloid dendritic cells drives progression of relapsing experimental autoimmune encephalomyelitis. Ann. N. Y. Acad. Sci..

[12] Constantinescu C.S., Farooqi N., O’Brien K., Gran B. (2011). Experimental autoimmune encephalomyelitis (EAE) as a model for multiple sclerosis (MS). Br. J. Pharmacol..

[13] McCarthy D.P., Richards M.H., Miller S.D. (2012). Mouse models of multiple sclerosis: Experimental autoimmune encephalomyelitis and Theiler’s virus-induced demyelinating disease. Methods Mol. Biol..

[14] Kipp M., Nyamoya S., Hochstrasser T., Amor S. (2017). Multiple sclerosis animal models: A clinical and histopathological perspective. Brain Pathol..

[15] Birmpili D., Charmarke Askar I., Bigaut K., Bagnard D. (2022). The Translatability of Multiple Sclerosis Animal Models for Biomarkers Discovery and Their Clinical Use. Int. J. Mol. Sci..

[16] Momtazmanesh S., Shobeiri P., Saghazadeh A., Teunissen C.E., Burman J., Szalardy L., Klivenyi P., Bartos A., Fernandes A., Rezaei N. (2021). Neuronal and glial CSF biomarkers in multiple sclerosis: A systematic review and meta-analysis. Rev. Neurosci..

[17] Jahan-Abad A.J., Karima S., Shateri S., Baram S.M., Rajaei S., Morteza-Zadeh P., Borhani-Haghighi M., Salari A.A., Nikzamir A., Gorji A. (2020). Serum pro-inflammatory and anti-inflammatory cytokines and the pathogenesis of experimental autoimmune encephalomyelitis. Neuropathology.

[18] Kuenz B., Deisenhammer F., Berger T., Reindl M. (2007). Diagnostic biomarkers in multiple sclerosis. Expert. Opin. Med. Diagn..

[19] Göbel K., Ruck T., Meuth S.G. (2018). Cytokine signaling in multiple sclerosis: Lost in translation. Mult. Scler. J..

[20] Imitola J., Chitnis T., Khoury S.J. (2005). Cytokines in multiple sclerosis: From bench to bedside. Pharmacol. Ther..

[21] Palle P., Monaghan K.L., Milne S.M., Wan E.C.K. (2017). Cytokine Signaling in Multiple Sclerosis and Its Therapeutic Applications. Med. Sci..

[22] Aharoni R., Eilam R., Lerner S., Shavit-Stein E., Dori A., Chapman J., Arnon R. (2021). Neuroprotective Effect of Glatiramer Acetate on Neurofilament Light Chain Leakage and Glutamate Excess in an Animal Model of Multiple Sclerosis. Int. J. Mol. Sci..

[23] Gnanapavan S., Grant D., Pryce G., Jackson S., Baker D., Giovannoni G. (2012). Neurofilament a biomarker of neurodegeneration in autoimmune encephalomyelitis. Autoimmunity.

[24] Brummer T., Schillner M., Steffen F., Kneilmann F., Wasser B., Uphaus T., Zipp F., Bittner S. (2023). Spatial transcriptomics and neurofilament light chain reveal changes in lesion patterns in murine autoimmune neuroinflammation. J. Neuroinflamm..

[25] Kuhle J., Barro C., Andreasson U., Derfuss T., Lindberg R., Sandelius Å., Liman V., Norgren N., Blennow K., Zetterberg H. (2016). Comparison of three analytical platforms for quantification of the neurofilament light chain in blood samples: ELISA, electrochemiluminescence immunoassay and Simoa. Clin. Chem. Lab. Med..

[26] Cohen L., Keegan A., Walt D.R. (2020). Single-Molecule Arrays for Ultrasensitive Detection of Blood-Based Biomarkers for Immunotherapy. Methods Mol. Biol..

[27] Lasseter H.C., Provost A.C., Chaby L.E., Daskalakis N.P., Haas M., Jeromin A. (2020). Cross-platform comparison of highly sensitive immunoassay technologies for cytokine markers: Platform performance in post-traumatic stress disorder and Parkinson’s disease. Cytokine X.

[28] Revendova K.Z., Zeman D., Bunganic R., Karasova K., Volny O., Bar M., Kusnierova P. (2022). Serum neurofilament levels in patients with multiple sclerosis: A comparison of SIMOA and high sensitivity ELISA assays and contributing factors to ELISA levels. Mult. Scler. Relat. Disord..

[29] Pafiti A., Krashias G., Tzartos J., Tzartos S., Stergiou C., Gaglia E., Smoleski I., Christodoulou C., Pantzaris M., Lambrianides A. (2023). A Comparison of Two Analytical Approaches for the Quantification of Neurofilament Light Chain, a Biomarker of Axonal Damage in Multiple Sclerosis. Int. J. Mol. Sci..

[30] Dong R., Yi N., Jiang D. (2024). Advances in single molecule arrays (SIMOA) for ultra-sensitive detection of biomolecules. Talanta.

[31] Poisson L.M., Suhail H., Singh J., Datta I., Denic A., Labuzek K., Hoda M.N., Shankar A., Kumar A., Cerghet M. (2015). Untargeted Plasma Metabolomics Identifies Endogenous Metabolite with Drug-like Properties in Chronic Animal Model of Multiple Sclerosis. J. Biol. Chem..

[32] Porro C., Cianciulli A., Panaro M.A. (2020). The Regulatory Role of IL-10 in Neurodegenerative Diseases. Biomolecules.

[33] Rissin D.M., Kan C.W., Campbell T.G., Howes S.C., Fournier D.R., Song L., Piech T., Patel P.P., Chang L., Rivnak A.J. (2010). Single-molecule enzyme-linked immunosorbent assay detects serum proteins at subfemtomolar concentrations. Nat. Biotechnol..

[34] Arroyo Pereiro P., Muñoz-Vendrell A., León Moreno I., Bau L., Matas E., Romero-Pinel L., Yélamos A.M., Yélamos S.M., Andrés-Benito P. (2023). Baseline serum neurofilament light chain levels differentiate aggressive from benign forms of relapsing–remitting multiple sclerosis: A 20-year follows-up cohort. J. Neurol..

[35] Yang Z., Wang K.K. (2015). Glial fibrillary acidic protein: From intermediate filament assembly and gliosis to neurobiomarker. Trends Neurosci..

[36] Barro C., Benkert P., Disanto G., Tsagkas C., Amann M., Naegelin Y., Leppert D., Gobbi C., Granziera C., Yaldizli O. (2018). Serum neurofilament as a predictor of disease worsening and brain and spinal cord atrophy in multiple sclerosis. Brain.

[37] Bridel C., van Wieringen W.N., Zetterberg H., Tijms B.M., Teunissen C.E., The NFL Group (2019). Diagnostic Value of Cerebrospinal Fluid Neurofilament Light Protein in Neurology: A Systematic Review and Meta-analysis. JAMA Neurol..

[38] Uher T., McComb M., Galkin S., Srpova B., Oechtering J., Barro C., Tyblova M., Bergsland N., Krasensky J., Dwyer M. (2021). Neurofilament levels are associated with blood–brain barrier integrity, lymphocyte extravasation, and risk factors following the first demyelinating event in multiple sclerosis. Mult. Scler. J..

[39] Wagner C.A., Roqué P.J., Goverman J.M. (2020). Pathogenic T cell cytokines in multiple sclerosis. J. Exp. Med..

[40] Bhise V., Dhib-Jalbut S. (2016). Further understanding of the immunopathology of multiple sclerosis: Impact on future treatments. Expert. Rev. Clin. Immunol..

[41] Chen Y.C., Yang X., Miao L., Liu Z.G., Li W., Zhao Z.X., Sun X.J., Jiang G.X., Chen S.D., Cheng Q. (2012). Serum level of interleukin-6 in Chinese patients with multiple sclerosis. J. Neuroimmunol..

[42] Chen Y.C., Chen S.D., Miao L., Liu Z.G., Li W., Zhao Z.X., Sun X.J., Jiang G.X., Cheng Q. (2012). Serum levels of interleukin (IL)-18, IL-23 and IL-17 in Chinese patients with multiple sclerosis. J. Neuroimmunol..

[43] Martins T.B., Rose J.W., Jaskowski T.D., Wilson A.R., Husebye D., Seraj H.S., Hill H.R. (2011). Analysis of proinflammatory and anti-inflammatory cytokine serum concentrations in patients with multiple sclerosis by using a multiplexed immunoassay. Am. J. Clin. Pathol..

[44] Vandenbark A.A., Morgan E., Bartholomew R., Bourdette D., Whitham R., Carlo D., Gold D., Hashim G., Offner H. (2001). TCR peptide therapy in human autoimmune diseases. Neurochem. Res..

[45] Bettelli E., Das M.P., Howard E.D., Weiner H.L., Sobel R.A., Kuchroo V.K. (1998). IL-10 is critical in the regulation of autoimmune encephalomyelitis as demonstrated by studies of IL-10- and IL-4-deficient and transgenic mice. J. Immunol..

[46] Cua D.J., Groux H., Hinton D.R., Stohlman S.A., Coffman R.L. (1999). Transgenic interleukin 10 prevents induction of experimental autoimmune encephalomyelitis. J. Exp. Med..

[47] Zahoor I., Nematullah M., Ahmed M.E., Fatma M., Sajad M., Ayasolla K., Cerghet M., Palaniyandi S., Ceci V., Carrera G. (2025). Maresin-1 promotes neuroprotection and modulates metabolic and inflammatory responses in disease-associated cell types in preclinical models of Multiple Sclerosis. J. Biol. Chem..

[48] Zahoor I., Suhail H., Datta I., Ahmed M.E., Poisson L.M., Waters J., Rashid F., Bin R., Singh J., Cerghet M. (2022). Blood-based untargeted metabolomics in relapsing-remitting multiple sclerosis revealed the testable therapeutic target. Proc. Natl. Acad. Sci. USA.

[49] Zahoor I., Waters J., Datta I., Cerghet M., Poisson L., Rattan R., Giri S. (2022). Predicting Disease Progression in Multiple Sclerosis by using a Combination of Highly Sensitive Single Molecule Array Technology (SIMOA) and Untargeted Metabolomics. Mult. Scler..

[50] Mangalam A., Poisson L., Nemutlu E., Datta I., Denic A., Dzeja P., Rodriguez M., Rattan R., Giri S. (2013). Profile of Circulatory Metabolites in a Relapsing-remitting Animal Model of Multiple Sclerosis using Global Metabolomics. J. Clin. Cell Immunol..

[51] Mangalam A.K., Rattan R., Suhail H., Singh J., Hoda M.N., Deshpande M., Fulzele S., Denic A., Shridhar V., Kumar A. (2016). AMP-Activated Protein Kinase Suppresses Autoimmune Central Nervous System Disease by Regulating M1-Type Macrophage-Th17 Axis. J. Immunol..

